# “I believe in my ancestors, and I participate and believe:” negotiating identity, tradition, and HIV-related health among SGM communities in the Eastern Cape

**DOI:** 10.1016/j.ssmqr.2026.100715

**Published:** 2026-02-12

**Authors:** Kelly Rutt, Lindsey de Vos, Aphiwe Metula, Remco P.H. Peters, Cikizwa Bongo, Leigh Ann van der Merwe, Joseph Daniels

**Affiliations:** a David Geffen School of Medicine, UCLA, USA; b Desmond Tutu Health Foundation, South Africa; c Foundation for Professional Development, South Africa; d Department of Medical Microbiology, University of Pretoria, Pretoria, South Africa; e Edson College of Nursing Innovation, Arizona State University, USA; f Social, Health and Empowerment Feminist Collective of Transgender Women of Africa, Buffalo City, Eastern Cape, South Africa

## Abstract

**Background:**

Sexual and gender minority (SGM) individuals in South Africa’s Eastern Cape face dual challenges navigating progressive constitutional protections alongside persistent cultural conservatism. Traditional Xhosa practices, including initiation schools (Ulwaluko) and ceremonial rituals, enforce rigid gender roles that conflict with SGM identities. With HIV prevalence reaching 49.5 % among men who have sex with men yet only 25.7 % achieving viral suppression, understanding how SGM individuals negotiate cultural traditions while managing HIV remains critically understudied.

**Methods:**

This qualitative study explored post-intervention experiences of 31 SGM individuals living with HIV in the Eastern Cape following participation in the SOAR (Speaking Out & Allying Relationships) intervention. Semi-structured interviews, guided by Social Cognitive Theory, were conducted in participants’ preferred language by trained local interviewers. Thematic analysis through the Minority Stress Model examined four domains: traditional practices, family dynamics, community perceptions, and intervention impact.

**Results:**

Participants demonstrated resilience through selective participation in cultural practices, balancing ancestral reverence with identity authenticity. Family acceptance was often conditional, tied to economic contributions or heteronormative expectations. Health communication barriers persisted due to stigma and traditional beliefs linking HIV to moral failing. SOAR enhanced participants’ status-sharing confidence, treatment adherence, and skills for navigating cultural tensions, though community stigma remained pronounced in rural areas.

**Conclusion:**

SGM individuals exhibit remarkable adaptability in negotiating intersecting cultural, familial, and health challenges. While SOAR effectively builds individual resilience and HIV management skills, findings underscore the need for multi-level interventions combining skill-building with community mobilization and cultural sensitization to address structural barriers in traditional contexts.

## 1. Introduction

**Fig. 1.**
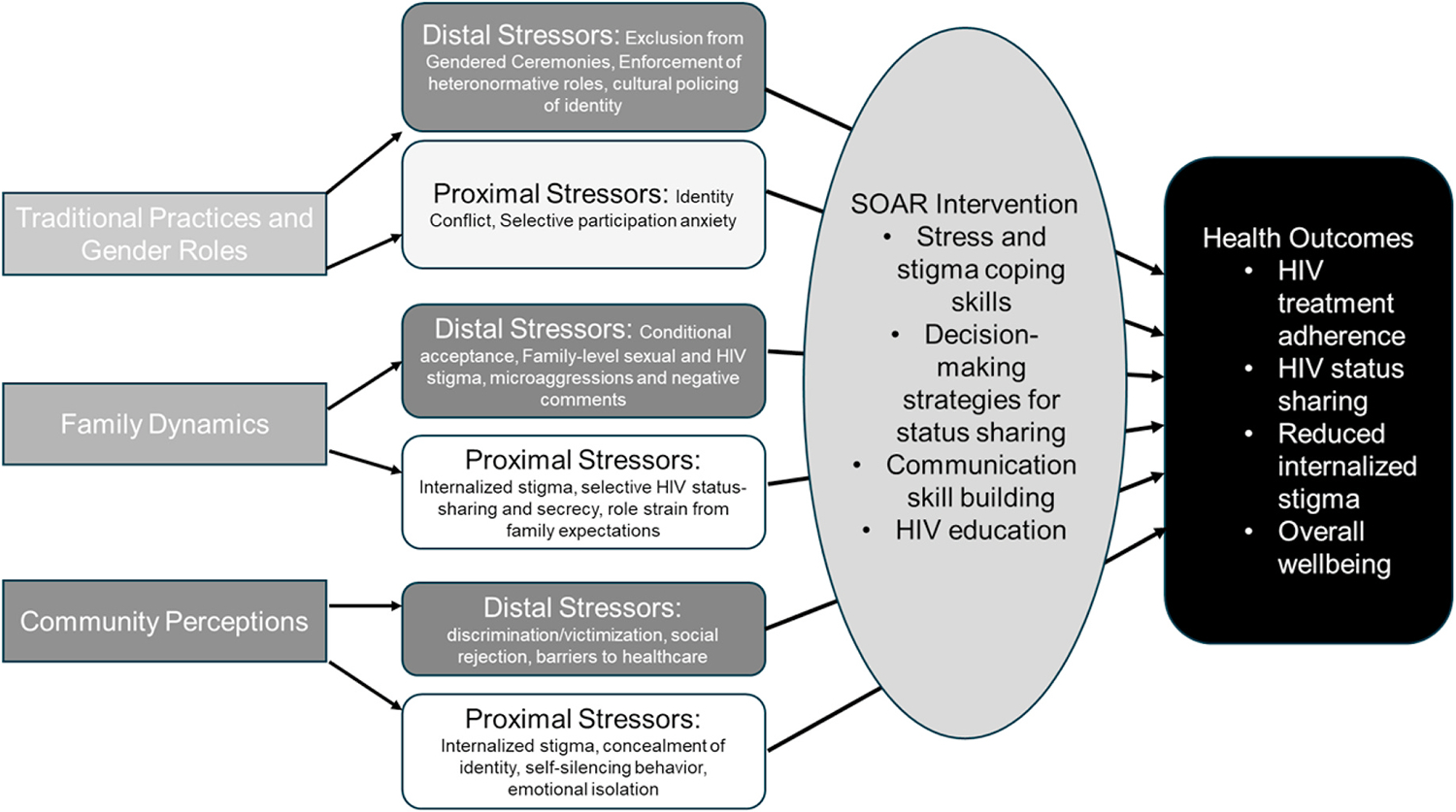
SGM and the SOAR intervention through the minority stress model.

The global HIV epidemic continues to pose significant health challenges, with approximately 40 million people living with HIV (PLWH) by 2023. () This widespread prevalence underscores the need for effective prevention and management strategies, including retention in care. In South Africa, the situation is particularly dire, with high HIV-related mortality rates, such as 77.9 deaths per 100,000 population. (South Africa) Despite increased access to antiretroviral therapy (ART), environmental factors such as stigma continue to discourage status sharing among PLHIV, leading many to avoid status sharing to prevent social rejection and stigma. The benefits of status sharing are often outweighed by these fears, making status-keeping a common strategy for maintaining privacy and avoiding negative consequences in many societies (Mokgatle and Madiba, 2023; Madiba and Mokgatle, 2016; Pantelic et al., 2020; Gittings et al.). (see Table 1, Fig. 1)

The Eastern Cape province (ECP), with strong adherence to traditional practices, faces considerable complexities in addressing HIV due to cultural and societal barriers (Gittings et al.; Kangethe, 2023). The ECP is marked by significant challenges, experiencing high prevalence rates due to a combination of structural, cultural, and health system barriers. In 2022, the South African HIV Prevalence, Incidence and Behavior Survey reported that the ECP had the fourth highest HIV prevalence nationally at 13.7 % across all ages, with prevalence peaking at 34.8 % among individuals aged 40–44 years (Smith, 2024). Rural areas, such as those in the ECP, are disproportionately affected by HIV due to limited healthcare infrastructure, stigma, and traditional practices that may hinder prevention and treatment efforts (Kangethe, 2023).

South Africa presents a dual landscape of progressive legal protections for LGBTQ + individuals and persistent cultural conservatism that perpetuates stigma and exclusion (Mthembu, 2023; Reygan and Lynette, 2014). The country’s 1996 Constitution was the first globally to explicitly prohibit discrimination based on sexual orientation, enshrining equality for sexual and gender minorities (SGM) (Cock, 2003). South Africa’s National Strategic Plan (NSP) for HIV, TB, and STIs 2023–2028 is centered on reducing barriers to health and social services, with a strong emphasis on equity, human rights, and the inclusion of key and priority populations, including sexual and gender minorities. (South African National AIDS Council) The NSP explicitly commits to people-centered, community-led interventions and prioritizes breaking down stigma, discrimination, and harmful gender norms that impede access to care for SGM. However, this legal and policy framework starkly contrasts with deeply rooted cultural traditions, such as Xhosa initiation schools (Ulwaluko, ritual male circumcision) and Imbeleko ceremonies (ritual introduction of a newborn child to their ancestors and the wider family), which enforce rigid gender roles and patriarchal norms (Brown, 2024a; Mfecane, 2020). These practices, while central to community identity, often marginalize SGM individuals, particularly trans women and gay men, by conflating biological sex with socially mandated gender performance. This tension is compounded by South Africa’s HIV epidemic, where SGM communities face disproportionate burdens: HIV prevalence among men who have sex with men (MSM) reaches 49.5 % in some regions, yet only 25.7 % achieve viral suppression due to structural barriers (e.g. lack of adequate access to healthcare, economic disparity, transportation limitations) and intersectional stigma (Daniels et al., 2023, 2025; Johnson and Dorrington, 2024). For transwomen, the HIV prevalence is approximately 46 % in the Buffalo City Metro of the ECP (van der Merwe et al., 2004).

While evidence-based interventions such as Healthy Relationships and couple-based HIV counseling and testing have demonstrated efficacy in improving HIV status disclosure, critical gaps remain for sexual and gender minority populations in culturally conservative settings where traditional practices enforce rigid gender norms that conflict with non-heteronormative identities (Daniels et al., 2022; Kalichman et al., 2001; Lin et al., 2016). The SOAR (Speaking Out and Allying Relationships) intervention addresses this gap by adapting evidence-based disclosure strategies f gay, bisexual, and transgender individuals living with HIV (LWH) in South Africa’s Eastern Cape, equipping participants with skills to manage stress, navigate status sharing, and improve treatment adherence via videoconference delivery (Daniels et al., 2022, 2023, 2025). the intervention’s efficacy in mitigating minority stressors and shaping health outcomes within a sociocultural context where constitutional rights and traditional norms remain in conflict. (Mthembu, 2023; Reygan and Lynette, 2014).

## 2. Methods

### 2.1. Study overview and setting

Using a descriptive qualitative approach, this study explores how sexual and gender minority (SGM) individuals in South Africa navigate cultural traditions, familial relationships, and HIV management following participation in the SOAR intervention. Semi-structured in-depth interviews, grounded in Social Cognitive Theory and analyzed through the Minority Stress Model framework, were designed to capture nuanced accounts of how MSM, gay, bisexual, and trans participants negotiate gendered cultural practices and health communication post-intervention. Data were collected between November 2024 and February 2025 in the ECP, a region marked by strong adherence to Xhosa traditions (Brown, 2024a; Kasa, 2024; Mfecane, 2016) alongside high HIV prevalence (Gittings et al.; Kangethe, 2023).

### 2.2. SOAR intervention

The SOAR intervention was developed through a human-centered design approach, adapted from the Healthy Relationships model for SGM individuals LWH in South Africa (Daniels et al., 2022, 2023, 2025; Kalichman et al., 2001). Core components of SOAR include: (1) building coping skills to manage HIV-related stress and sexual risk contexts, (2) enhancing decision-making strategies for HIV status sharing to partners, and (3) establishing safer sexual practices through role-plays and scenarios tailored to South African SGM experiences. Delivered via videoconference to improve accessibility, the intervention comprises five, 90-min group sessions and three 30-min booster sessions, integrating culturally relevant media and skill-building activities to address stigma, treatment adherence, and relationship dynamics with partners and family members. Grounded in Social Cognitive Theory, SOAR emphasizes self-efficacy, environmental interactions, and self-regulation to empower participants in navigating status sharing, ART adherence, and sexuality and gender affirmation within stigmatizing contexts. The adaptation process involved in-depth interviews with SGM communities to ensure alignment with local cultural norms and healthcare barriers, particularly for trans women and gay/bisexual men in the ECP (Daniels et al., 2023, 2025; Kalichman et al., 2001).

### 2.3. Study sample and treatment

A purposive sample of 31 SGM individuals LWH was recruited through the research team’s partnerships with LGBTQ + -affirming clinics and community organizations in the ECP (Patterson et al., 2017; Smalley et al., 2017, p. 455). Inclusion criteria for the main study (n = 21) required participants to (1) identify as MSM, gay, bisexual, trans, or gender-nonconforming, (2) be aged ≥18 years, and (3) have completed the SOAR intervention, a 6-week program addressing stress management, HIV status sharing strategies, and ART adherence. Additionally, participants (n = 10) were interviewed who specifically identified as trans women and completed the trans-specific SOAR intervention sub-study. It is also important to note that 5 of the 21 initial participants identified as MSM or gay upon enrollment and subsequently identified as transwomen during the study. Individuals were recruited based on established guidelines for qualitative research, with a focus on achieving data saturation rather than statistical representativeness (Hennink and Kaiser, 2022; Saunders et al., 2018). Recruitment materials were disseminated through clinic staff and community partners familiar with the target population, and snowball sampling was also used to reach SGM individuals through existing participant networks. The sample size was determined through an iterative approach, where data collection and preliminary analysis occurred concurrently until theoretical saturation was reached (Hennink and Kaiser, 2022; Saunders et al., 2018).

### 2.4. Data collection

31 Semi-structured in-depth interviews (IDIs) of approximately 1 h were conducted post-SOAR in participants’ preferred language (isiXhosa or English) by trained South African interviewers who were fluent in both. The interview guide was pilot-tested with community members from the research team and refined based on their feedback to ensure cultural appropriateness and clarity. Interviews probed the following domains: community perception, family dynamics, health management, and the impact of the SOAR intervention on their status sharing and adherence behaviors. 21 follow-up interviews were then conducted to explore additional themes uncovered after the initial analysis in the domain of traditional cultural practices. These shorter interviews, approximately 30 min in length, focused specifically on cultural ceremony participation, family negotiations surrounding traditional roles, and the intersection of ancestral beliefs with SGM identity. Saturation was assessed throughout the analysis process by examining whether new interviews generated novel codes or themes or primarily replicated previously identified patterns (Saunders et al., 2018; Willig and Rogers, 2017, p. 665). Regular team meetings monitored saturation progress and ensured consensus on when sufficient depth and breadth of data had been achieved. All interviews were audio-recorded, transcribed verbatim, and translated into English. Transcripts were anonymized to protect confidentiality.

### 2.5. Theoretical framework

This study is anchored in SCT (Bandura, 1989) and the Minority Stress Model (Meyer, 2003a). SCT guided the development of SOAR’s core components, emphasizing observational learning, self-efficacy, and reciprocal determinism between personal agency and environmental influences. The intervention’s focus on skill-building (e.g., stress management, HIV status sharing strategies) reflects SCT’s principles, leveraging role-playing and peer modeling to enhance participants’ confidence in navigating health decisions and cultural expectations (Daniels et al., 2023).

Minority Stress Model provides a critical lens to examine how systemic stigma, cultural traditions, and internalized prejudice shape SGM individuals’ health behaviors and ceremonial participation. The distinction between distal stressors and proximal stressors aligns with participant accounts of negotiating identity within Xhosa traditions. By integrating SCT’s emphasis on adaptive coping and the Minority Stress Model’s focus on structural inequities, this framework illuminates how SOAR’s skill-building strategies mediate the impact of minority stress, enabling participants to assert agency in culturally constrained contexts. Together, these theories contextualize the interplay of personal resilience, cultural negotiation, and systemic barriers in SGM health outcomes.

### 2.6. Data analysis

Data analysis was conducted using a rigorous thematic approach to capture the nuanced experiences of SGM individuals navigating traditional practices, family dynamics, and health management in the ECP following participation in the SOAR intervention (Braun and Clarke, 2014; Willig and Rogers, 2017, p. 665). Team members engaged in extensive memoing during a first read of the transcripts, capturing preliminary interpretations, emergent patterns, theoretical connections, and establishing the four domains for this analysis (Neale, 2016; Saldana, 2021, pp. 1–440). All transcripts were then coded by multiple team members (JD, LD, AM, KR) through an iterative coding process to ensure reliability and minimize individual bias (Saldana, 2021, pp. 1–440). These independently developed codes were then compared in regular team meetings to reach consensus and create a unified codebook organized around the four determined domains: traditional practices and gender roles, family dynamics, community perceptions, and intervention impact. In subsequent coding rounds, two or more team members coded the same interviews to establish inter-coder reliability and refine code definitions. Emergent patterns included participants’ strategies for selective cultural participation, the conditional nature of familial acceptance, persistent health communication barriers linked to HIV-related stigma, and the protective effects of the SOAR intervention on status-sharing confidence and treatment adherence. Themes were iteratively refined as new data were reviewed, with team discussions used to resolve coding disagreements and ensure congruence between the data and our analytical framework (Willig and Rogers, 2017, p. 665). This collaborative approach enhanced the validity and rigor of our findings.

### 2.7. Ethics

Ethical approval for this study was obtained from the XXX Review Board (189/2022) with reliance by the Institutional Review Board at XXX (STUDY00014539). The research was conducted with authorization from the relevant provincial and district departments of health, as well as local health research committees. All participants provided written informed consent after receiving a thorough explanation of the study procedures and the nature of the in-depth interviews. To acknowledge their time and contribution, participants were offered a snack and received R150 (approximately $10 USD) as compensation.

## 3. Results

The results of this analysis are organized around four key domains that emerged from participants’ narratives. First, Traditional Practices and Gender Roles explores how cultural ceremonies and expectations shape, challenge, or support the identities of SGM individuals in the ECP. The domain of Family Dynamics examines the complexities of familial acceptance, conditional support, and the negotiation of status sharing within households marked by both tradition and change. Community Perceptions captures the broader social environment, highlighting experiences of stigma, acceptance, and the influence of rural-urban divides on the daily lives of SGM people. Finally, SOAR Intervention Impact assesses how participation in the videoconference-based SOAR program influenced participants’ abilities to manage these tensions, creating action plans to manage the stresses involved in status-sharing decisions and treatment adherence. This assessment will provide insight into the potential of tailored interventions to foster resilience and agency within this context.

### 3.1. Traditional practices and gender roles

#### 3.1.1. Conflict with identity

Participants consistently described personal internal conflicts between their SGM identities and culturally enforced gender expectations, particularly during initiation schools and ceremonies like Imbeleko. One participant articulated this tension, noting that while ancestral traditions provided spiritual grounding, “I believe practicing them brings me closer to my ancestors,” (M, age 28, 4 years LWH) initiation teachings about the role of a man directly clashed with his identity as a feminine gay man, forcing him to perform hypermasculine roles in male-only cultural communal spaces like the kraal. Other participants described participation in certain practices centered on pleasing parents and other family members.

I think for me, even going through the initiation process feels like I was forced because as much as I went through it myself, I was also doing it in respect of my parents, as I was not aware at that time that my rights were also being violated in a way. (M, age 36, 8 years LWH)

There are things that are meant to be done by a man and things that are meant to be done by women, and those things are disadvantageous to me because they don’t play a role in my life or do something important to my life for me to say that I cannot be a gay person because culture and tradition doesn’t allow it. An example I can make is that a boy has to go to an initiation school, but as a gay person, we don’t see any reason for us to go to initiation school and be taught how to be a man because we know that we are gays … They are a challenge because you are seen as a man in the community, whereas you identify yourself as gay. As a result, once you are in a space with people, traditionally, a man must go to initiation school, take care of outside chores like gardening and painting the houses. Personally, I don’t believe in those things, and I don’t conform to them. NBNGage 27, <1-year LWH)

Some participants chose to participate in cultural rituals to maintain a sense of connection with their heritage and to honor family expectations, viewing these practices as a way to strengthen ties with their ancestors and their faith. Through their involvement, they remained connected to their religious devotion, a sense of belonging, and familial acceptance, even as they navigated the challenges.

I’m someone who likes to balance things. I believe in God, and I also believe in culture and tradition. As a result, I believe in my ancestors, and I participate and believe in the traditional practices that are done at home because they are the ones that allow me to live my life properly without any issues.

(TW, age 25, 3 years LWH)

At home, we do traditional practices, and we believe in them and our cultural beliefs. And for me, as a gay person, they don’t affect me in any way because my family tells me everything about these traditional practices and what is expected of me … I do them because they are done by my family, they believe in them, and I grew up seeing them live by tradition, cultural norms, and values.

(M, age 26, 1 year, LWH)

Some participants navigated discomfort in male-dominated ceremonial tasks, acknowledging an awkwardness when their peers do not understand their sexuality. Yet, they still comply with gendered chores and practices to maintain cultural belonging.

As I grow older, I now realize that these traditional practices also challenge my identity because traditionally I am regarded as a man and during these traditional ceremonies. I am expected to sit with men in the kraal and do everything with them, even though I am a bottom gay person, meaning that I am a bit more on the feminine side. However, I do men’s chores during these ceremonies, and I don’t mind that because it’s how I grew up, but it is awkward for me to be in the presence of these men. It’s awkward because they don’t understand my sexuality, and it’s uncomfortable to be in their presence, knowing how traditional they are.

(M, aged 28, 4 years LWH)

I see traditional practices or perspectives as being important in our lives because we grew up in families who believe and conform to these practices, so I don’t see them as being a bad thing. I even went to initiation school because my family wanted me to go, as it is a tradition that is done at home. So, I just went there for them … For me, as a gay person, these traditional practices are a challenge or disadvantage to me because they are judging my sexuality. As much as they are important, to me, they are a challenge because they go against the life I am living. There are things that I do that these practices do not support …. I feel like traditional practices or perspectives challenge my identity in the community because they don’t support the fact that I am gay, they are judgmental towards my sexuality and there are things that traditionally or culturally we are not supposed to do, but as a gay person I want to do them, such as dating other men because traditionally a man is supposed to date a woman.

(W, age 28, <1-year LWH)

Conversely, some participants’ experiences contrasted with these narratives, as they participated freely in ceremonies and events without harassment, suggesting acceptance in some traditional contexts.

I would say they have a positive influence on my relationship with my family because at home, they believe in tradition and ancestors; as a result, they like doing traditional practices and ceremonies to appease the ancestors. When we get together with my family, they tell me everything about the tradition, and I do what I need to do as per tradition. One thing I like about my family is that they know and have accepted my sexuality, so they support me, and I also do what they expect of me.

(M, age 26,1 year LWH)

For some participants, being gay and being a man are experienced as separate but coexisting aspects of their identity. They described how they performed traditional male roles during cultural ceremonies, such as sitting with men in the kraal or taking on men’s chores, not as a denial of their sexuality, but as an affirmation of their masculinity and cultural belonging. This distinction highlights that masculinity is not inherently tied to heterosexuality; rather, gay men can embody and express masculine traits while also embracing their sexual orientation.

Yes, they (challenges) are there irrespective of your sexuality, but it goes with acceptance from the family. For example, I’m going to my rural area tomorrow because one of my brothers is going to the initiation school, and I’m part of that process. The fact that they know that I’m gay doesn’t mean that they must exclude me from such things. I partake in the preparations and the process of initiation because I’ve also gone through it, even though I’m gay. Yes, some people do question me about being gay and being involved in such ceremonies, but my sexuality doesn’t change the fact that I am a man.

(M, age 38, 14 years LWH)

Collectively, these accounts reveal how rigid gender norms in cultural practices perpetuated identity conflicts, with participants expressing the need to balance ancestral reverence with self-affirmation.

#### 3.1.2. Selective participation

Many SGM individuals in the ECP selectively participate in cultural traditions based on their gender identity and sexual orientation, navigating a complex landscape of acceptance and exclusion.

For me, traditional practices have no influence on how I see myself, even though when there’s a cultural ceremony happening there’re those rules that a man should sit where other men are sitting, As result I don’t just attend any traditional ceremony that is happen around my neighborhood because I’m trying to avoid such rules and I only attend those that I’m invited to because I know I will sit with my friends wherever I’ll be seated.

(M, age 30, 1 year LWH)

If the traditional practice/ceremony is being done at my home, everything just goes well, and I’d be fine but when they it’s being done by a neighbor. I don’t attend because of thing that are said by the men of the society and that doesn’t sit well with me, it makes me feel judged for being transwoman and they don’t understand who I am.

(TW, age 33, 16 years LWH)

### 3.2. Family dynamics

#### 3.2.1. Conditional acceptance

Conditional acceptance of participants’ sexuality by family members was a recurring theme among SGM participants, who often described being tolerated or included by relatives as long as they fulfilled certain roles or contributed economically to the household. While some families offered practical support or refrained from outright rejection, this acceptance was frequently contingent on participants concealing aspects of their sexual or gender identity, adhering to traditional expectations, or providing financial assistance, highlighting the precariousness and limitations of familial support in a context marked by pervasive cultural conservatism in the ECP.

I think they (my family) have accepted me but not fully. They just accepted it because I am working, and they expect me to support them … I would say (traditional practices) influence my relationship with my family in a bad way because at home we believe in different traditions. Some of my family members are Christians, and others believe in traditional practices and ancestors, just like me. As a result, that causes a bit of conflict in my family.

(W, age 28, <1 year, LWH)

Even when some participants were accepted on the surface by their families, they often reported being expected to conform to traditional male roles and practices, such as participating in gendered ceremonies or fulfilling male-specific duties. At the same time, they had to navigate subtle or overt negative comments and microaggressions from family members, reflecting persistent underlying tensions and a lack of full affirmation for their SGM identities.

She (my aunt) supports me in everything, she knows about my sexuality. She knows about how I live. My aunt is the only one who knows about all my problems, and she helps me deal with them. She’s the one who did a burial for my mom, and she also sent me to initiation school and did Umgidi for me. Umgidi is a traditional ceremony done to celebrate someone coming back from the mountain (initiation school) for male circumcision. My aunt is my supporter. She’s also the one who paid for my education. In my mother’s side of the family, I haven’t disclosed my status to anyone.

(TW, age 27, 9 years, LWH)

Bearing in mind that family is not only your parents, but also everyone you are related to. My parents understand some of the things that never sit well with me. But, there are people in the family who say we expect this person to do this and in this manner. That’s where the challenge comes because maybe I don’t see the necessity of me doing all of that, but on the other hand, you have an elder telling you that you must do this.

(M, age 38, 14 years LWH)

They’re always against it (my sexuality), obviously because of the cultural norms and all that. But from my side, me being well informed about my sexuality, I know how to handle my family, I know what to say in a polite manner without fighting, so that they fully understand who I am and can stand up for me when someone says something bad to me or about me.

(M, age 26, <1 year, LWH)

#### 3.2.2. Family support

Full familial support emerged as a powerful protective factor for SGM individuals, enabling them to withstand and navigate community-level stigma and discrimination. When families offered affirmation and acceptance, participants described increased confidence, psychological well-being, and the resilience needed to maintain their identity and health behaviors despite external pressures.

My sisters and brothers know that I’m a trans woman, and these traditions don’t have an influence on my relationship with them. My family supports me. As a result, when I go outside, I don’t care what people say because I have full support at home. You find that some people don’t say anything to harass you when they know that your family supports you.

(049)

They don’t exclude me from any of the proceedings, so I feel supported. Even if there’s something that needs to be done by the men of the family, I get included in it, as much as they know my sexuality.

(M, age 30, 1 year LWH)

I am open about my sexuality. My family knows and they support me, and I do participate in all traditional practices that are done at home despite my sexuality. There hasn’t been anything traditional or cultural that has caused a challenge in my sexuality or identity. Even in the community as well, I haven’t experienced a challenge … One thing I like about my family is that they know and have accepted my sexuality, so they support me, and I also do what they expect of me.

(M, age 26, 1 year LWH)

In contrast, the absence of familial support left participants more vulnerable to the negative effects of community stigma, often resulting in heightened isolation, distress, and challenges in maintaining both their identity and HIV-related health behaviors. They describe the difficulty of facing obstacles and rejection without support, saying “even when we face these challenges, we face them alone (W, age 28, <1 year LWH).”

I am struggling with depression … It’s my relationship with my dad; sometimes we don’t talk. There are times where you need to talk to a parent and share what’s troubling you, but I cannot do that with my dad. I talk to my aunt, but I don’t want to trouble her with everything because she has her own problems, and she has to be there for her daughter. I would also love for my father to be there for me and support me … At home, I don’t like to socialize with my parents. I solve my own problems and deal with my stresses alone. Sometimes it’s hard because I want my parents to be there for me, but they are not. So, I use what I learnt in the study to manage my stress and use coping skills.

(TW, age 27, 9 years LWH)

I take my treatment at 7:00am, and it gets tense when I have to take it because I fear that they (my family) can see me. I don’t want to lie, I am not taking my treatment properly, and I am not ready to disclose my status to my family. If I could have my own place to stay, maybe in years to come, I would disclose my status to them, knowing that I have my own place. For now, it’s not easy, sometimes I don’t take treatment for maybe 3 days.

(TW, age 25, 3 years LWH)

#### 3.2.3. Health communication barriers

Health communication barriers within families were a significant challenge for SGM individuals, often resulting in limited or avoided discussions about HIV and related health behaviors. Many participants described how their voices were dismissed or deemed unimportant in family HIV conversations, with elders or relatives either actively avoiding the topic or invalidating the perspectives of SGM members, reinforcing secrecy and further isolating them from vital support and information.

At home I am not see as being important because of my sexuality so it’s not easy for me to talk to my family with issues like HIV because they don’t value me.

(TW, age 28, <1 year LWH)

Firstly, people still stigmatize people, and as soon as they know about your status, there are things that they feel like you shouldn’t be doing because of your status. I’m not using my local clinic since I don’t want to expose my status, so I am using the government clinics in East London because in our local clinic facilities people will know when who’ve come to collect your ART because we’ll be told to go to that consulting room for HIV treatment, so then they because aware of your status from that and now I am not out about my status …. I think it’s discrimination like maybe one is ashamed to go to the clinic

(M, age 27, 3 years LWH)

P: Yes, we do, but not all of us. A lot of gays don’t want to go to clinics and check their HIV status, they would rather stay sick … I think it’s the fear of being judged and losing their loved ones because of their status.

(TW, age 27, 9 years LWH)

Many participants describe their families having trouble discussing HIV due to deeply rooted cultural and traditional beliefs that frame the disease as a source of shame, moral failing, and stigma. These barriers are reinforced by religious and spiritual values, which can contribute to blaming or shaming those living with HIV and discourage open dialogue about prevention, testing, or treatment. Beliefs in traditional healers, who suggest they can cure HIV, further complicate efforts to seek biomedical care.

Even the fact that you’re transgender … they (family) will say this is demonic, hence you’re HIV+, it’s because you’re using private parts that were not meant to be used in this manner.

(M, age 36, 8 years LWH)

Uhmm, one traditional practice that I’ve heard concerning HIV was that there is a Traditional healer who is able to heal and cure HIV, but I know that’s impossible, so I don’t believe in that and I don’t take it to mind.

(TW, age 30, 19 years LWH)

People still stigmatize people, and as soon as they know about your status, there are things that they feel like you shouldn’t be doing because of your status.

(M, age 38, 14 years LWH)

When it comes to talking about HIV with my family, I am free to talk about it because they support me, especially the ones that I stay with now. It would be difficult to talk about it with my family, that’s in the villages, because they don’t have enough knowledge about HIV.

(TW, age 31, 9 years, LWH)

### 3.3. Community perceptions - stigma vs. acceptance

Community perceptions played a pivotal role in shaping the daily experiences of SGM individuals in the ECP, influencing their sense of safety, belonging, and openness about their identities, as well as their healthcare choices. While some reported experiences of acceptance and inclusion, such as being welcomed at traditional ceremonies or feeling respected by peers and neighbors, many more recounted persistent stigma and discrimination that permeated daily life. Community members often policed gender norms through gossip, name-calling, and social exclusion, with labels like “wannabe woman” or accusations of violating cultural traditions serving as tools of social control.

Well, from my community, I was never judged or discriminated against because of my sexuality. They always make me feel welcome, and they always make sure that I always stay as who I am. I do attend ceremonies, any traditional ceremony, and I sit with whoever I want to sit with, be it the men or girls, and I don’t get harassed for that. Even when I went through initiation myself, it was a fun experience because my peers then were fine with me, some knew my sexuality, and some didn’t, but I was a fun experience I really enjoyed it. I didn’t experience any difficulty while there.

(M, age 26, <1 year LWH)

Obviously, with traditional norms and expectations, a man is supposed to have a wife and be masculine, so all of that does implicate the way that one must live their lives as a gay man. I think before it was something I used to be concerned about, but as the years passed, with me being comfortable in my stance and knowing who I am, it’s something that doesn’t really affect me anymore. I have passed that.

(M, age 29, 4 years LWH)

I think stigma from society because most of the people in the Eastern Cape go through initiation school and all that, so it’s instilled in them that a man is supposed to behave in a certain way, they are supposed to do this and that. Also, from what I have witnessed a lot of them are afraid to access the department of health facilities like your local clinics when they have like certain STIs, especially when it’s an STI that is related with an anus and whatnot they fear being discriminated against in the local clinics because a lot of the nurses there are still not embracing or not as welcoming to clients that are of the LGBTQI community.

(M, age 29, 4 years LWH)

### 3.4. SOAR intervention impact

The findings illustrate the SOAR intervention’s impacts across key domains. Participants describe enhanced ability to navigate cultural, familial, and health challenges. SOAR equipped participants with coping strategies to manage stress from conflicting cultural expectations (e.g., reconciling initiation school teachings with LGBTQ + identities) while fostering agency to engage in ceremonies on their own terms, selectively. Participants expressed that the intervention improved communication skills for HIV status sharing and treatment discussions, though familial acceptance remained conditional and tied to economic contributions in many cases. SOAR provided participants with the skills to articulate their choices regarding engagement with gendered ceremonies and cultural expectations in a way that balanced respect for tradition with affirmation of their sexual and gender identities, fostering greater agency and confidence in navigating these complex dynamics. For example, one participant shared, “What I remember is that I was struggling with how I must disclose to my peers, but in the study I have learned how to disclose and how to take a person’s response to my status sharing, whether it’s negative or positive, it is fine” (M, age 34, 18 years LWH). Another noted, “After SOAR, things changed a lot. The people to whom I disclosed my HIV status and sexuality accepted the situation. It was not easy, but they did” (M, age 28, 4 years LWH). These concise statements highlight the concrete ways in which the SOAR intervention fostered resilience and acceptance among participants, fulfilling its aim to support both skill-building and psychosocial well-being.

SOAR was described as bolstering resilience against stigma through peer support networks and skill-building, enabling participants to advocate for their identities in both traditional and urban settings. At the same time, they acknowledged that rural areas continued to pose significant barriers despite these gains.

Before I joined the study, people would try and say negative things about sexuality and being HIV positive and say a lot of bad things. There’s also a stigma that is attached towards the gay sexuality, people tend to believe that when you’re gay, then that means you’re HIV positive. If you’re gay, it’s like you deserve to be HIV positive without people even knowing your HIV status, they just assume. After being in the SOAR study, I learnt that some things in life are not there to keep you, and they are not there to teach you something better. If you were to listen to everything that people say, you won’t get anywhere, you might end up being suicidal. After the SOAR study, I can now stand better for myself and make good choices for myself without having to ask people what to do, like I would do before, because I would ask people how to handle certain things.

(M, age 23, 2 years LWH)

## 4. Discussion

This study illuminates the complex interplay of minority stress, cultural traditions, and health outcomes among SGM individuals in South Africa’s ECP, using Meyer’s Minority Stress Model (Meyer, 2003b, 2015). Findings reveal how structural stigma (distal stressors) and internalized prejudice (proximal stressors) intersect to shape HIV management and cultural negotiation, with resilience strategies and interventions like SOAR mediating these effects (Sun et al., 2022). Applying the Minority Stress Model as a theoretical lens, this study demonstrates how SGM individuals navigate intersecting pressures from traditional practices, family expectations, and community attitudes while managing HIV. The findings reveal that rigid gender norms, particularly those enforced during initiation schools and ancestral ceremonies, create significant distal stressors by excluding or marginalizing SGM individuals who do not conform to heteronormative ideals. These experiences echo the broader literature showing that traditional gender roles in South Africa are deeply entrenched and often policed through social sanctions, making it difficult for those who transgress these norms, such as gay men and trans women, to fully participate in cultural life without facing judgment or exclusion. In response, many SGM individuals engage in selective participation, adapting their involvement in rituals or choosing which roles to assume to maintain both cultural belonging and authentic self-expression.

Distal stressors rooted in cultural norms manifested as exclusion from gendered ceremonial roles, including trans women barred from Imbeleko rituals (Brown, 2024b) and gay men pressured to perform hypermasculine tasks (Mashabane and Henderson, 2020; Mfecane, 2016). These practices, central to Xhosa identity, enforced heteronormative expectations that may conflict with SGM identities, perpetuating internalized stigma (proximal stress) and limiting health communication (da luz Scherf, 2024). Our findings echo but also extend prior scholarship, where many participants described experiencing tension between cultural expectations and their sexual or gender identities (Mkhize and Mthembu, 2023; Reygan and Lynette, 2014). This study found that some SGM individuals actively negotiate their involvement in rituals, demonstrating resilience by adapting their participation rather than withdrawing entirely, which nuances the typically binary portrayals in earlier literature (Cock, 2003; Mfecane, 2012). These adaptive strategies, such as selectively attending ceremonies or redefining the meaning of their roles parallel reports from similar populations and also suggest greater agency and subtle acts of self-affirmation than have been previously documented. This process is often accompanied by discomfort and emotional labor, reflecting resilience and ongoing negotiation to balance belonging with self-expression, especially in rural, traditional communities with strong heteronormativity enforcement. Findings further illustrate that, in specific contexts, familial support can facilitate acceptance within traditional settings, which contrasts with the literature that emphasizes unconditional exclusion (Mfecane, 2016). Thus, our results suggest a more complex and varied landscape of cultural negotiation for SGM individuals in the Eastern Cape.

Results highlight the dual role of families as both sources of support and sources of proximal stress. Some SGM individuals experience full familial acceptance and benefit from the protective effects, while others describe conditional acceptance tied to economic contributions or adherence to traditional expectations. These findings align with research showing family support can buffer community stigma, but it is frequently precarious and contingent (Lin et al., 2022; Wang et al., 2022). Families mediated minority stress through conditional acceptance, often tied to economic contributions or heteronormative roles (Kavanaugh et al., 2020; Mkhize and Mthembu, 2023). Some participants leveraged familial support to buffer stigma, while others faced dismissal in health discussions, with elders framing HIV as “demonic”. These dynamics reflect the “dual reality” of South African families as both protective and oppressive (Müller, 2020), underscoring a need for interventions training cultural mediators, like aunts and sisters, to bridge intergenerational HIV communication gaps. Health communication barriers, such as the dismissal of SGM voices in HIV discussions due to perceived unimportance, isolate individuals and reinforce secrecy, underscoring the need for interventions strengthening intergenerational communication and leveraging respected family mediators. Sharing of HIV status often relied on trusted relatives, yet patriarchal norms limited open dialogue, reinforcing secrecy and isolation (Madiba and Mokgatle, 2016; Mokgatle and Madiba, 2023). This precarious support system highlights the intersection of economic dependency and cultural conservatism, where SGM individuals navigate familial tolerance while muting aspects of their identity.

Structural stigma persists for both SGM and PLWH, especially in rural areas, where traditional leaders policed gendered spaces (Mthembu, 2023; Letsoalo et al., 2021; Moruri-Silo and Mushonga) and healthcare providers stigmatized anal health issues as “promiscuity” (Mange et al., 2022; Ramalepe and Maake, 2025; Ravele et al., 2025). Internalized stigma appears in self-silencing, such as avoiding clinic visits post-ulwaluko, while other participants report growing visibility through LGBTQ + events. National surveys show over 70 % of South Africans agree PLHIV experience stigma and are treated differently, and approximately half would not engage in relationships or care for family members LWH (Mokgatle and Madiba, 2023). Yet grassroots activism fostered pockets of solidarity. Gossip and labeling as social control underscores the tension between constitutional protections and lived realities (Cock, 2003).

Judgment and scrutiny often lead SGM individuals to self-silence, avoiding open discussions about identity or health to protect themselves from ridicule or rejection. This duality involves constant negotiation of self-presentation, selective status sharing, and managing stigma from family and community, all while striving for well-being and access to care (Harper et al., 2013; Pamoso et al., 2024; Rothmann, 2018). Fear of judgement or outing influences healthcare choices, with some avoiding local clinics or more anonymous care to keep HIV status or gender identity private. Despite these challenges, some communities offer solidarity and support, with allies among peers or within social circles. Community mobilization and communication skill-building may shift gender norms and amplify acceptance through inclusive events and programs (Ferguson et al., 2023; Leddy et al., 2021).

Many South African communities rely on traditional healers, with over 80 % of rural populations seeking their services for both physical and psychological needs (Zuma et al., 2018). This cultural reliance shapes illness interpretation, with some viewing HIV as spiritual or ancestral rather than biomedical, causing diagnosis and treatment delays or use of traditional remedies over antiretroviral therapy (Maleke et al., 2019a). Growing recognition exists to bridge tradition and biomedical treatment, as some traditional healers are now train to refer clients for HIV testing and collaborate with clinics, highlighting potential for culturally sensitive integration that respects both healing systems while promoting effective HIV care (Audet et al., 2022; Maleke et al., 2019b).

The SOAR intervention demonstrated considerable impact across the interrelated domains of family dynamics, community perceptions, and traditional practices. SOAR’s skill-building components reduced proximal stressors by enhancing status-sharing confidence (Daniels et al., 2022, 2023) and treatment adherence through peer support (Kalichman et al., 2005). Within family systems, SOAR provided participants with tools to manage communication about HIV status and sexual identity, fostering improved dialogue and, in some cases, greater acceptance within the household. While family support is widely recognized as a critical buffer against stigma and a predictor of positive health outcomes, the intervention also highlighted the persistent reality of conditional acceptance. SOAR’s peer support and skill-building components equipped participants to respond resiliently to distal stressors, such as community-level stigma, fostering a sense of agency and self-efficacy in navigating hostile environments. Participants credited SOAR with improving communication strategies, such as using “pill partners” to reinforce ART adherence, mirroring findings from Healthy Relationships adaptations (Daniels et al., 2023). However, gaps persist as participants face cultural barriers, and trans women require tailored content (Auerbach et al., 2020). These limitations echo broader critiques of top-down interventions that fail to address intersectional stigma (Mokgatle and Madiba, 2023). The study highlights the limitations of individual-level interventions in the face of entrenched stigma, pointing to the need for multi-level approaches that combine SOAR’s skill-building with community mobilization, advocacy, and ongoing engagement with traditional leaders to amplify its positive effects.

### 4.1. Implications for policy and practice

This study underscores the need for interventions like SOAR that address social and interpersonal challenges. SOAR builds skills for HIV status sharing, stress management, and treatment adherence, empowering participants to navigate cultural traditions, family expectations, and community stigma. To maximize impact, SOAR and similar programs must be integrated with efforts to sensitize traditional leaders and communities about gender and sexual identity diversity, fostering inclusive cultural participation. Family-centered approaches can leverage respected mediators like aunts or grandmothers to facilitate open communication about HIV and sexuality, challenging conditional acceptance that hinges on economic or heteronormative role adherence. Community mobilization, modeled on peer-led programs, can reduce stigma and promote acceptance while addressing barriers faced by rural and trans populations. SOAR’s virtual delivery should ensure accessibility, incorporating education about long-acting injectable ART, gender-affirming care, and resources for those avoiding clinic care due to stigma. Embedding SOAR within a holistic framework of cultural integration, family engagement, and community mobilization can help bridge the gap between South Africa’s progressive legal protections and SGM individuals’ lived realities.

### 4.2. Limitations and future directions

South African data collectors, as members of local communities, brought nuanced cultural insight and linguistic expertise to the research, while the non-South African authors contributed academic expertise and experience, necessitating ongoing reflexivity to ensure that local knowledge, lived experiences, and power dynamics were foregrounded and respected throughout the research process. This study was co-designed with SGM communities to ensure cultural relevance, sustainability, and genuine empowerment in the ongoing effort to bridge the gap between tradition, identity, and health. While it offers valuable insights into the nuanced realities of SGM communities in a culturally conservative context, its small sample size and cross-sectional design limit the generalizability of the findings. Future research should prioritize longitudinal studies (cohorts) to explore how evolving traditions and interventions, such as SOAR, impact health outcomes, the retention of coping skills, and the long-term development of resilience among SGM individuals.

## 5. Conclusion

This study explores how SGM individuals in the Eastern Cape actively negotiate traditional cultural practices and familial dynamics in the context of HIV-related health challenges. By examining these adaptive strategies, the findings contribute new insights into the intersection of culture, stigma, and identity, and highlight the importance of multi-level interventions tailored to both individual and structural barriers. SGM individuals in the ECP demonstrate remarkable capacity for adaptability as they negotiate the intersecting demands of identity, tradition, and health. Their lived experiences highlight not only the challenges posed by rigid cultural norms and pervasive HIV stigma but also the creative strategies they employ to assert their identities and maintain well-being. Future research should further investigate opportunities to strengthen culturally responsive support for SGM communities within health systems.

## Figures and Tables

**Table 1:** Socio-demographics of interviewed SOAR participants.

Characteristic (n/%)	Total (N = 31)
Age range	
20–24	5 (16.1 %)
25–29	14 (45.2 %)
30–37	9 (29 %)
38–43	3 (9.7 %%)
Sexual identity	
Homosexual/gay	28 (90.3 %)
Bisexual	0 (0.0 %)
Men who have sex with men (MSM)	2 (6.5 %)
Heterosexual	1 (3.2 %)
Gender identity	
Man (M)	15 (48.4 %)
Trans woman (TW)	8 (25.8)
Non-binary/gender-non-conforming (NBGN)	4 (12.9 %)
Woman (W)	3 (9.7 %)
Did Not ID (DNI)	1 (3.2)
Years living with HIV	
Newly diagnosed	1 (3.2 %)
< 1 year	6 (19.4 %)
1–4 years	13 (41.9 %)
5–8 years	4 (12.9 %)
9–20 years	7 (22.6 %)
Partner relationship type	
In a monogamous relationship	15 (48.4 %)
In a non-monogamous or open relationship	3 (9.7 %)
In a polyamorous relationship	2 (6.5 %)
Friends with benefits	4 (12.9 %)
Fuck buddies/it's just sex	5 (16.1 %)
Other	1 (3.2 %)
Missing	1 (3.2 %)
Reported partner's HIV status	
Positive	7 (22.5 %)
Negative	12 (38.75 %)
Don't know/missing	12 (38.75 %)
